# Supplementary material for: Deepening Physical Exercise Intervention Protocols for Older People with Sarcopenia Following Establishment of the EWGSOP2 Consensus: A Systematic Review
Source: Geriatrics (Basel). 2025 Jul 4;10(4):91. doi: 10.3390/geriatrics10040091 (PMC12285934; doi:10.3390/geriatrics10040091)
Supplement: Supplementary file 1 [file geriatrics-10-00091-s001.zip › Supplementary Tables S1-S3.pdf]

Supplementary Materials

Table S1. Summary of pre- and post- outcomes and within-/between-group significance across included studies.

Authors (Year)	Variables		Preintervention (SD)	Postintervention (SD)	Significant Difference	
					Within Groups	Between Groups
Courel-Ibáñez, J (2022)	Muscle strength	Handgrip (kg)	IG: 20.1 (6.1)<br>CG: 16.3 (8.4)	IG: 19.0 (2.3)<br>CG: 20.8 (2.1)	IG: □<br>CG: ■	Post: □
		Handgrip/Bm (kg/kg)	IG: -<br>CG: -	IG: 0.27 (0)<br>CG: 0.29 (0)	IG: □<br>CG: ■	Post: □
	Physical performance	SPPB (points)	IG: 4.1 (3.1)<br>CG: 4.4 (2.8)	IG: 8.7 (2.3)<br>CG: 6.6 (2.1)	IG: ■<br>CG: ■	Post: □
		Timed up-and-go (s)	IG: 23.0 (9.1)<br>CG: 29.2 (18.9)	IG: 18.4 (5.8)<br>CG: 19.4 (5)	IG: □<br>CG: □	Post: □
		Gait speed 6m (m/s)	IG: 0.49 (0.2)<br>CG: 0.48 (0.2)	IG: 0.77 (0.2)<br>CG: 0.71 (0.2)	IG: ■<br>CG: ■	Post: □
		Sit-to-stand (s)	IG: -<br>CG: -	IG: 10.9 (2.7)<br>CG: 12.4 (2.7)	IG: ■<br>CG: ■	Post: □
		Sit-to-stand MPV (m/s)	IG: -<br>CG: -	IG: 0.42 (0.1)<br>CG: 0.46 (0.2)	IG: □<br>CG: □	Post: □
De Sá Souza, H (2022)	Other variables	Sleep latency (min)	IG: 25.86 (20.2)<br>CG: 21.59 (15.7)	IG: 16.09 (5.2)<br>CG: 29.98 (16.1)	IG: □<br>CG: □	Post: ■
		REM sleep latency (min)	IG: 108.53 (73.2)<br>CG: 86.21 (44.3)	IG: 117.10 (70.9)<br>CG: 103.00 (45.4)	IG: □<br>CG: □	Post: □
		Total sleep time (min)	IG: 310.00 (61.2)<br>CG: 342.00 (90)	IG: 298.86 (84.9)<br>CG: 325.14 (66.6)	IG: □<br>CG: □	Post: □
		Sleep efficiency (%)	IG: 67.05 (13.5)<br>CG: 72.32 (18.7)	IG: 67.92 (20.1)<br>CG: 69.49 (13.3)	IG: □<br>CG: □	Post: □
		Sleep stage - N1	IG: 20.62 (15.8)<br>CG: 10.27 (4.4)	IG: 22.31 (21.3)<br>CG: 10.23 (3.8)	IG: □<br>CG: □	Post: ■
		Sleep stage - N2	IG: 37.34 (11.9)<br>CG: 39.57 (11.8)	IG: 37.37 (8.7)<br>CG: 42.60 (10.6)	IG: □<br>CG: □	Post: □
		Sleep stage - N3	IG: 25.55 (9.8)<br>CG: 32.18 (11.6)	IG: 30.25 (11.2)<br>CG: 27.80 (9.1)	IG: □<br>CG: □	Post: □
		Wake after sleep onset	IG: 128.42 (58.2)<br>CG: 80.21 (46.5)	IG: 131.21 (94.9)<br>CG: 98.63 (40.8)	IG: □<br>CG: □	Post: □
		Apnea/hour (n/h)	IG: 16.82 (14.1)<br>CG: 9.68 (4.5)	IG: 7.37 (7.5)<br>CG: 13.29 (7.7)	IG: ■<br>CG: □	Post: □
		Apnea hypopnea index (n/h)	IG: 14.08 (15.6)<br>CG: 15.20 (13.1)	IG: 9.37 (13.7)<br>CG: 15.13 (15.9)	IG: □<br>CG: □	Post: ■
		SPO2<90% (min)	IG: 6.84 (10.3)<br>CG: 7.02 (10.7)	IG: 5.00 (8.7)<br>CG: 6.55 (11)	IG: □<br>CG: □	Post: □
		Testosterone (ng/dL)	IG: 329.02 (298)<br>CG: 127.00 (211.6)	IG: 322.16 (317.7)<br>CG: 130.65 (226.4)	IG: □<br>CG: □	Pre: ■<br>Post: ■
		Growth hormone (ng/mL)	IG: 1.21 (1.3)<br>CG: 1.95 (2.4)	IG: 1.26 (1.2)<br>CG: 1.11 (1.3)	IG: □<br>CG: □	Post: □
		Insulin-like growth factor 1 (ng/mL)	IG: 7.76 (20.5)<br>CG: 12.27 (28.2)	IG: 7.33 (20.5)<br>CG: 14.60 (28.2)	IG: □<br>CG: □	Post: □
		Cortisol (ug/dL)	IG: 10.05 (3.2)<br>CG: 12.29 (3.1)	IG: 11.29 (2.6)<br>CG: 13.40 (2.1)	IG: □<br>CG: □	Post: □
		Tumor necrosis factor -α (pg/mL)	IG: 4.40 (0.3)<br>CG: 4.30 (0.4)	IG: 4.41 (0.3)<br>CG: 4.17 (0.3)	IG: □<br>CG: □	Post: □
		Interleukin-6 (pg/mL)	IG: 2.92 (1.1)<br>CG: 2.88 (0.7)	IG: 3.29 (1.7)<br>CG: 2.56 (0.6)	IG: □<br>CG: □	Post: □
		Interleukin-10 (pg/mL)	IG: 2.13 (0.8)<br>CG: 1.96 (0.6)	IG: 2.51 (1)<br>CG: 2.26 (0.9)	IG: ■<br>CG: □	Post: □
		Interleukin-1 RA (ng/mL)	IG: 0.93 (0)<br>CG: 0.95 (0.1)	IG: 0.99 (0.1)<br>CG: 0.94 (0.1)	IG: ■<br>CG: □	Post: □
Flor-Rufino, C (2023)a	Muscle strength	Average - maximum isometric knee exten-	IG: 16.15 (6.6)<br>CG: 13.89 (5.1)	IG: 22.11 (7.3)<br>CG: 15.80 (7.2)	IG: ■<br>CG: □	Post: ■

		sion				
		Dynamic muscle strength – knee extension (kg)	IG: 3.68 (3.7)<br>CG: 2.75 (3.2)	IG: 11.32 (6.5)<br>CG: 2.59 (3)	IG: ■<br>CG: □	Post: ■
		Dynamic muscle strength – leg press (kg)	IG: 58.61 (21)<br>CG: 59.87 (17.9)	IG: 82.54 (19.6)<br>CG: 58.13 (21.2)	IG: ■<br>CG: □	Post: ■
	Body composition	Skeletal muscle index (kg/m^2)	IG: 5.63 (0.8)<br>CG: 5.85 (0.6)	IG: 6.02 (0.7)<br>CG: 5.79 (0.8)	IG: ■<br>CG: □	Post: ■
		Muscle mass (kg)	IG: 35.20 (4)<br>CG: 37.12 (3)	IG: 36.30 (4.1)<br>CG: 36.43 (4.1)	IG: ■<br>CG: □	Post: ■
		Fat mass (kg)	IG: 22.53 (7.1)<br>CG: 25.04 (5.2)	IG: 21.29 (6.4)<br>CG: 25.89 (6.6)	IG: □<br>CG: □	Post: ■
		Body weight (kg)	IG: 59.63 (9.6)<br>CG: 63.55 (8.2)	IG: 59.50 (9.7)<br>CG: 63.44 (9.1)	IG: □<br>CG: □	Post: □
		BMI (kg/m^2)	IG: 26.18 (4.2)<br>CG: 27.69 (2.9)	IG: 26.02 (4.3)<br>CG: 27.52 (3.3)	IG: □<br>CG: □	Post: □
	Physical performance	Gait speed (m/s)	IG: 0.79 (0.2)<br>CG: 0.71 (0.2)	IG: 0.85 (0.2)<br>CG: 0.76 (0.2)	IG: □<br>CG: □	Post: □
		SPPB total (points)	IG: 7.57 (3.1)<br>CG: 7.72 (2.3)	IG: 8.67 (3.5)<br>CG: 8.17 (2.9)	IG: ■<br>CG: □	Post: □
		SPPB balance (points)	IG: 2.70 (1.1)<br>CG: 3.06 (1.2)	IG: 3.10 (1.3)<br>CG: 3.17 (1.2)	IG: □<br>CG: □	Post: □
		SPPB walk (points)	IG: 2.85 (0.9)<br>CG: 2.44 (0.8)	IG: 3.00 (0.9)<br>CG: 2.50 (1)	IG: □<br>CG: □	Post: □
		SPPB sit to stand (points)	IG: 2.35 (1.5)<br>CG: 2.22 (1.3)	IG: 3.00 (1.6)<br>CG: 2.44 (1.4)	IG: ■<br>CG: □	Post: □
**Flor-Rufino, C (2023)b**	Other variables	Maximal inspiratory pressure (cm H_2O)	IG: 46.35 (20.2)<br>CG: 49.11 (16.7)	IG: 50.60 (20)<br>CG: 49.33 (14.2)	IG: □<br>CG: □	Post: □
		Maximal expliratory pressure (cm H_2O)	IG: 76.30 (22.9)<br>CG: 88.33 (22.8)	IG: 88.25 (31.5)<br>CG: 86.44 (24.9)	IG: □<br>CG: □	Post: ■
		FVC (L)	IG: 1.91 (0.6)<br>CG: 2.02 (0.6)	IG: 1.82 (0.5)<br>CG: 1.85 (0.4)	IG: □<br>CG: ■	Post: □
		FEV_1 (L)	IG: 1.49 (0.4)<br>CG: 1.60 (0.5)	IG: 1.45 (0.4)<br>CG: 1.47 (0.4)	IG: □<br>CG: ■	Post: □
		FEV_1/FVC (%)	IG: 79.77 (13.5)<br>CG: 79.24 (6.5)	IG: 80.53 (12.2)<br>CG: 79.19 (7.7)	IG: □<br>CG: □	Post: □
		FEV_{25-75} (L/s)	IG: 1.40 (0.7)<br>CG: 1.58 (0.7)	IG: 1.57 (1)<br>CG: 1.53 (0.8)	IG: □<br>CG: □	Post: □
		PEF (L/s)	IG: 3.77 (0.8)<br>CG: 3.90 (1.8)	IG: 3.99 (1.1)<br>CG: 3.75 (1.5)	IG: □<br>CG: □	Post: □
		Health related (points)	IG: 67.25 (15.3)<br>CG: 66.94 (19.3)	IG: 73.00 (16.9)<br>CG: 61.11 (18.2)	IG: □<br>CG: □	Post: □
		Health related Index (points)	IG: 0.68 (0.2)<br>CG: 0.65 (0.2)	IG: 0.69 (0.2)<br>CG: 0.58 (0.2)	IG: □<br>CG: □	Post: □
**Guo, H (2024)**	Muscle strength	Handgrip (Kg)	IG TCSG: 19.28 (2.7)<br>IG STG: 19.73 (3.1)<br>CG: 18.72 (3.5)	IG TCSG: 20.60 (1.5)<br>IG STG: 21.35 (2.3)<br>CG: 17.67 (3.1)	IG TCSG: ■<br>IG STG: ■<br>CG: □	Post: ■
	Body composition	Relative skeletal muscle mass index (kg/m2)	IG TCSG: 5.10 (0.9)<br>IG STG: 4.84 (1.2)<br>CG: 5.32 (1.2)	IG TCSG: 6.22 (0.9)<br>IG STG: 6.05 (1.3)<br>CG: 5.24 (1.3)	IG TCSG: ■<br>IG STG: ■<br>CG: □	Post: ■
		Skeletal muscle intramuscular fat (cm2)	IG TCSG: 14.91 (1.6)<br>IG STG: 14.73 (1.4)<br>CG: 14.28 (1.2)	IG TCSG: 15.15 (1.5)<br>IG STG: 15.30 (1.6)<br>CG: 15.03 (1.4)	IG TCSG: □<br>IG STG: □<br>CG: □	Post: □
		Skeletal muscle area (cm2)	IG TCSG: 77.01 (4.4)<br>IG STG: 75.79 (4.8)<br>CG: 77.63 (5.8)	IG TCSG: 81.83 (3.8)<br>IG STG: 78.64 (6.4)<br>CG: 77.06 (4.5)	IG TCSG: ■<br>IG STG: ■<br>CG: □	Post: ■
		Skeletal muscle density (Hounsfield units)	IG TCSG: 32.30 (1.8)<br>IG STG: 32.64 (3)<br>CG: 32.69 (3.7)	IG TSCG: 34.60 (1.9)<br>IG STG: 34.72 (2.8)<br>CG: 32.44 (3.3)	IG TCSG: ■<br>IG STG: ■<br>CG: □	Post: ■
		Skeletal muscle intramuscular density (Hounsfield units)	IG TCSG: -65.14 (4.2)<br>IG STG: -64.01 (5.4)<br>CG: -64.21(5.7)	IG TCSG: -65.35 (3.7)<br>IG STG: -64.48 (4.7)<br>CG: -64.68 (6.1)	IG TCSG: □<br>IG STG: □<br>CG: □	Post: □

Hsiao-Ting, T (2021)	Muscle strength	Upper limb endurance (times)	IG: 4.96 (4.3)<br>CG: 6.14 (4.4)	IG: 7.81 (4.7)<br>CG: 4.65 (4.3)	IG: ■<br>CG: ■	Post: ■
		Lower limb endurance (times)	IG: 1.33 (2.6)<br>CG: 1.91 (4.1)	IG: 1.90 (3.3)<br>CG: 1.76 (3.8)	IG: □<br>CG: □	Post: ■
	Physical performance	Barthel Index (points)	IG: 50.70 (28.8)<br>CG: 51.84 (33.1)	IG: 60.96 (30)<br>CG: 46.18 (33.8)	IG: ■<br>CG: ■	Post: ■
	Other variables	Pulmonary Function (L/min)	IG: 142.28 (66.4)<br>CG: 172.25 (80.9)	IG: 177.50 (81)<br>CG: 156.27 (81.9)	IG: ■<br>CG: ■	Post: ■
		Lower Body Flexibility (cm)	IG: -21.38 (13.6)<br>CG: -26.25 (14.6)	IG: -16.17 (13.7)<br>CG: -29.79 (14.7)	IG: ■<br>CG: ■	Post: ■
		Shoulder Flexibility (º)	IG: -33.51 (18.9)<br>CG: -35.16 (20.7)	IG: -27.82 (18.3)<br>CG: -38.23 (20.1)	IG: ■<br>CG: ■	Post: ■
		Shoulder Flexion (º)	IG: 126.18 (19.4)<br>CG: 129.65 (18.2)	IG: 135.15 (17.6)<br>CG: 122.73 (22)	IG: ■<br>CG: ■	Post: ■
		Shoulder Abduction (º)	IG: 118.42 (21.4)<br>CG: 123.16 (23.4)	IG: 129.83 (20.7)<br>CG: 114.98 (25.3)	IG: ■<br>CG: ■	Post: ■
Ilke- Sen, E (2021)	Physical performance	Timed up and go (s)	IG: 12.5 (3.8)<br>CG: 13.7 (4.3)	IG: 11.4 (3.9)<br>CG: 14.3 (5.7)	IG: ■<br>CG: □	Post: ■
		6-minute walk test (m)	IG: 311.0 (105.9)<br>CG: 292.5 (115.5)	IG: 342.4 (110.8)<br>CG: 298.0 (129.0)	IG: ■<br>CG: □	Post: ■
		Berg Balance Scale (score)	IG: 42.8 (9.5)<br>CG: 41.6 (10.1)	IG: 47.4 (8.1)<br>CG: 41.8 (9.3)	IG: ■<br>CG: □	Post: ■
	Other variables	Quality of life (score)	IG: 4.8 (1.5)<br>CG: 4.7 (1.5)	IG: 3.9 (2.01)<br>CG: 4.7 (1.9)	IG: ■<br>CG: □	Post: ■
Liang, Y (2020)	Muscle strength	Handgrip (Kg)	IG: 16.3 (4.8)<br>CG: 14.9 (6.1)	IG: 20.5 (¶18.1-23.0)<br>CG: 16.3 (¶14.0-18.7)	IG: ■<br>CG: ■	Post: ■
	Physical performance	SPPB (points)	IG: 4.3 (1.4)<br>CG: 3.8 (1.8)	IG: 6.6 (¶5.7-7.5)<br>CG: 4.6 (¶4.0-5.2)	IG: ■<br>CG: ■	Post: ■
		Berg balance (score)	IG: 20.8 (15.1)<br>CG: 17.7 (10.2)	IG: 31.3 (¶26-36.5)<br>CG: 22 (¶18.1-25.9)	IG: ■<br>CG: ■	Post: □
		Time up and go (score)	IG: 23.4 (11.2)<br>CG: 40.4 (42.4)	IG: 18.5 (¶16.1-20.9)<br>CG: 31.7 (¶25.4-37.9)	IG: ■<br>CG: □	Post: □
		Barthel index (score)	IG: 72.3 (17.3)<br>CG: 64.5 (15.4)	IG: 81.8 (¶78.4-85.3)<br>CG: 70.8 (¶66.1-72.7)	IG: ■<br>CG: ■	Post: ■
Myong-Won, S (2021)	Muscle strength	Handgrip (kg)	IG: 20.8 (2.9)<br>CG: 18.6 (3.1)	IG: 24.3 (2.2)<br>CG: 17.3 (3.6)	IG: ■<br>CG: ■	Post: ■
		Maximum voluntary isometric contraction (N/m)	IG: 0112.4 (21.6)<br>CG: 97.6 (30.9)	IG: 123.5 (18.9)<br>CG: 93.7 (25.5)	IG: ■<br>CG: □	Post: □
		Relative maximum voluntary isometric contraction (N/m·kg)	IG: 209.9 (32.2)<br>CG: 195.4 (76.5)	IG: 231.8 (30.2)<br>CG: 186.6 (61.3)	IG: ■<br>CG: □	Post: □
	Body composition	Body weight (kg)	IG: 53.4 (4.4)<br>CG: 51.5 (5.6)	IG: 53.3 (4.4)<br>CG: 51.3 (5.9)	IG: □<br>CG: □	Post: □
		BMI (kg/m^2)	IG: 22.9 (2)<br>CG: 22.4 (1.5)	IG: 22.9 (2)<br>CG: 22.4 (1.6)	IG: □<br>CG: □	Post: □
		Waist circumference (cm)	IG: 76.2 (5.2)<br>CG: 77.4 (4.6)	IG: 76.1 (4.2)<br>CG: 78.4 (5.6)	IG: □<br>CG: □	Post: □
		Hip circumference (cm)	IG: 89.6 (4.4)<br>CG: 90.6 (4.3)	IG: 89.9 (3.7)<br>CG: 90.1 (4)	IG: □<br>CG: □	Post: □
		Waist hip ratios (points)	IG: 0.85 (0)<br>CG: 0.85 (0)	IG: 0.85 (0)<br>CG: 0.87 (0)	IG: □<br>CG: ■	Post: ■
		Fat mass (kg)	IG: 19.1 (3.1)<br>CG: 17.5 (3.6)	IG: 19.2 (3.2)<br>CG: 17.4 (3.8)	IG: □<br>CG: □	Post: □
		Fat-free mass (kg)	IG: 31.6 (2.1)<br>CG: 31.5 (2.6)	IG: 31.9 (1.9)<br>CG: 31.5 (2.9)	IG: □<br>CG: □	Post: □
		Appendicular skeletal muscle (kg)	IG: 12.3 (1)<br>CG: 12.4 (1)	IG: 12.4 (0.8)<br>CG: 12.2 (1.1)	IG: □<br>CG: □	Post: □
		Percent body fat (%)	IG: 36.3 (3.7)<br>CG: 34.4 (3.8)	IG: 36.3 (3.8)<br>CG: 34.3 (4.1)	IG: □<br>CG: □	Post: □
	Physical performance	30-s chair stand (n)	IG: 15.5 (4.5)<br>CG: 12.1 (3.1)	IG: 20.3 (5.2)<br>CG: 12.1 (3.1)	IG: ■<br>CG: □	Post: ■
		30-s arm curl (n)	IG: 16.3 (3.7)	IG: 21.2 (3.7)	IG: ■	Post: ■

			CG: 13.9 (3.3)	CG: 13.1 (2.6)	CG: □	
		Chair sit-and-reach (cm)	IG: 19.8 (7.1)<br>CG: 6.2 (10.6)	IG: 24.3 (7)<br>CG: 3.5 (10.1)	IG: ■<br>CG: ■	Post: ■
		8-foot up-and-go (s)	IG: 5.7 (0.5)<br>CG: 5.9 (0.7)	IG: 5.1 (0.4)<br>CG: 6.3 (0.8)	IG: ■<br>CG: ■	Post: ■
		2-min step test (n)	IG: 91.3 (11)<br>CG: 86.7 (13)	IG: 108.3 (11)<br>CG: 75.1 (19)	IG: ■<br>CG: ■	Post: ■
		Gait speed (m/s)	IG: 0.96 (0.1)<br>CG: 0.93 (0.1)	IG: 1.14 (0.1)<br>CG: 0.95 (0.1)	IG: ■<br>CG: □	Post: ■
	Other variables	Follistatin (pg/mL)	IG: 2113.75 (409.3)<br>CG: 2241.85 (669.9)	IG: 2652.85 (704.2)<br>CG: 2255.45 (564)	IG: ■<br>CG: □	Post: ■
		Growth differentiation factor-8 (pg/mL)	IG: 2294.43 (686.6)<br>CG: 1616.52 (650.7)	IG: 2193.11 (618.9)<br>CG: 1784.22 (529.7)	IG: □<br>CG: □	Post: □
		Growth differentiation factor-15 (pg/mL)	IG: 902.00 (406.9)<br>CG: 831.03 (262.8)	IG: 902.00 (406.9)<br>CG: 949.32 (326.7)	IG: □<br>CG: □	Post: □
		Activin A (pg/mL)	IG: 399.81 (93.6)<br>CG: 340.37 (61.6)	IG: 362.42 (76.8)<br>CG: 305.94 (53)	IG: □<br>CG: □	Post: □
**Sang-Jung, W (2019)**	Muscle mass	Fat-free mass (Kg)	IG: 32.3 (2.0)<br>CG: 33.0 (2.6)	IG: 33.4 (1.8)<br>CG: 32.8 (3.1)	IG: ■<br>CG: □	Post: ■
		Appendicular skeletal (Kg/m²)	IG: 5.3 (0.3)<br>CG: 5.2 (0.6)	IG: 5.3 (0.3)<br>CG: 5.2 (0.6)	IG: □<br>CG: □	Post: □
		Weight (Kg)	IG: 50.9 (4.7)<br>CG: 52.0 (5.1)	IG: 50.0 (4.8)<br>CG: 52.4 (4.7)	IG: ■<br>CG: □	Post: ■
		BMI (Kg/m²)	IG: 22.0 (1.3)<br>CG: 22.1 (1.2)	IG: 21.7 (1.2)<br>CG: 22.1 (1.4)	IG: ■<br>CG: □	Post: ■
		Fat mass (%)	IG: 33.6 (3.0)<br>CG: 33.9 (2.7)	IG: 31.8 (3.4)<br>CG: 35.1 (2.5)	IG: ■<br>CG: □	Post: ■

■: significant difference (significance determined at $p < 0.05$ unless otherwise noted), □: no significant difference, ¶: range interquartile.
5STS: five times sit-to-stand, BMI: body mass index, CG: control group, FEV: respiratory function, IG: intervention group, MPV: mean propulsive velocity, SD: standard deviation, SPPB: short physical performance battery, STG: Strength training, TCSG: Strength training hybrid group.

Table S2. Summary of participant adherence and dropouts across intervention groups of the included studies.

Authors (year)	Participant	Dropout	Adherence
Flor-Rufino, C (2023)a	24	4	83.3%
De Sá Souza, H (2022)	14	0	100%
Flor-Rufino, C (2023)b	24	4	83.3%
Hsiao-Ting, T (2021)	57	5	91.2%
Myong-Won, S (2021)	14	2	85.7%
Courel-Ibáñez, J (2022)	24	2	91.6%
Liang, Y (2020)	57	5	91.2%
Guo, H (2024)	NR	NR	NR
Sang Jung, W (2019)	13	0	100%
Ilke Sen, E (2021)	50	4	92.0%
NR: Not reported			

Table S3. Intervention's details.

Authors (year)	Intervention
Flor-Rufino, C (2023a)	The HIRT intervention consisted of two weekly 65 min sessions for six months, with a minimum recovery time of 72 h. Finally, participants in the HIRT group took part in up to 39 sessions (due to the university's vacation period). Each session consisted of three parts, starting with a 10-minute warm up, including joint mobility and postural control exercises. This was followed by a 45-min HIRT circuit, with six exercises to strengthen different muscle groups (two on the upper extremities, two on the trunk and two on the lower extremities). The present study focused only on the two lower extremity exercises (leg press and knee extension). Participants did three series of 10–15 repetitions until momentary failure. After a period of individualized progression in training, the load was set to at least 70 % of 1RM. Finally, during a 10- min cool down phase, participants did self-massage for myofascial release and stretching exercises. Attendance to the sessions was recorded daily, and adherence was categorized as high (>65 % of sessions), moderate (34–65 %), or low (<34 %). The CG did not receive any specific intervention for

	sarcopenia. Their participation in the trial was limited to telephone follow-ups to assess their general health status.
De Sá Souza, H (2022)	Eight exercises for large muscle groups used the Technogym Selection Pro® (Cesena, Itália) equipment, alternating upper and lower limbs (chest press, leg press, vertical traction, abdominal crunch, leg extension, arm curl, leg curl, and arm extension). RET was performed 3×/week for 12 weeks using a linear periodization model. In the first week, participants performed 1 set of 12 to 15 repetitions at 50% of 1RM. In the second week, they achieved 60% of 1RM, performing 2 sets of 10 to 12 reps. From the third week to the end of the protocol, 75% of 1RM was achieved (3 sets of maximum 8 reps). The intervals between series varied from 60 to 90 s. The training load was readjusted in the 6th training week using the 1RM test. The CTL group participated in weekly meetings with recommendations about lifestyle changes.
Flor-Rufino, C (2023b)	The HIRT intervention consisted of two 65-minute group sessions per week for six months. A total of 39 sessions were conducted, each consisting of three parts: a 10-minute warm-up, a 45-minute HIRT circuit, and a 10-minute cool-down. Participants in the HIRT circuit performed six strength exercises (two trunk, two arm and two leg exercises) to achieve integrated work and overall body improvement. Each exercise was performed at a 2:3 pace (2 s concentric action: 3 s eccentric action), with a two-minute rest between sets. The circuit had three sets per exercise and 10-15 repetitions per set, until failure, i.e., "the inability to perform further concentric contractions without significant changes in posture or repetition duration." After two weeks of individualized progression in training, the load was set to at least 70% of one repetition maximum (1RM), which was calculated with the submaximal equation. To maintain the correct progression of the load, every six sessions (three weeks) the 1RM of each participant was calculated. Thus, when the participant reached a new milestone in the 1RM assessment, the workload was adjusted to that value. CGs did not receive any specific intervention for sarcopenia but were encouraged to remain active. Session attendance was recorded daily, and adherence was categorized as adequate (> 70% of sessions) or low (≤ 70%).
Hsiao-Ting, T (2021)	Older adults with probable sarcopenia were taught to make a fist with a firm grip and to move the arms naturally to direct the fists to selected acupuncture points to stimulate the 14 meridians. Five fist styles were applied using different sides of the fist to strike. For example, the "phoenix fist" required seniors to first make a fist and then strike the acupoint with the palm side. The Vitality Acupunch exercise program was performed in a seated position and consisted of three phases: phase I qi and blood activation, phase II meridian tapping, and phase III body and mind relaxation. In phase I (warm-up), 5 slow and gentle movements were performed to loosen each joint. In phase II (exercise), 14 movements were performed to pierce the acupuncture points and vibrate the meridians to stimulate the circulation of qi and blood and strengthen cardiorespiratory endurance. In phase III (relaxation), 5 muscle relaxation movements with deep breathing were performed to calm the body. Each exercise session lasted 40 minutes and the Vitality Acupunch exercise program was performed three times a week for 6 consecutive months.
Myong-Won, S (2021)	The intervention program was conducted for 16 weeks from March to June in 2019.<br>The resistance training program was performed three times per week over sixteen weeks (48 sessions). Participants were asked to maintain their usual daily activities. Each training session included five minutes of warm-up, fifty minutes of the resistance exercise, and five minutes of cool-down. The weight-bearing exercises described by Watanabe et al. were performed for large muscle groups and further training for small muscle groups was done using an elastic band (Hygenic Corporation, Akron, OH, USA) resistance exercise program. The training load was increased by progressive overload and the OMNI resistance for active muscle scale (OMNI-RES AM, 0-extremely easy to 10-extremely hard) was used. The rest time between sets was 60 s.
Courel-Ibáñez, J (2022)	The Vivifrail tailored, multicomponent exercise program (http://vivifrail.com) was conducted to individually prescribe exercise for frail older adults, depending on their functional capacity. The training included 4 levels combining strength and power, balance, flexibility, and cardiovascular endurance exercises.
Liang, Y (2020)	The intervention group received a mixed exercise program including balance and resistance exercise. In each session, the participants received a light 5-min warm-up followed by 20 min of targeted balance training. Next, they were allotted a five-minute rest before another 20 min of resistance training. Each session ended with a 5-min cool-down that incorporated stretching. To be specific, the balance exercise program included: heel and toe raise and static balance in weeks 1–3; varied directional quick stepping in weeks 4–6; reaching and single-leg standing in weeks 7–9; heel to toe walking and complex cross-over stepping activities in weeks 10–12. The resistance exercise included leg press, leg extension and flexion, leg abduction and adduction, chest press, and seated row. The individual loads of resistance training were determined based on the strength test at the first intervention and at the 13th session. Resistance exercise was performed at 70–80% of one-repetition maximum, 3 sets of 8–12 repetitions each (with a 2-min rest between sets).<br><br>The control group engaged in a resistance exercise program. In each session, participants received a light 5-min warm-up first followed by 20 min of resistance training. Next, they were allotted a five-minute rest before another 20 min of resistance training. Each session also ended with a 5-min cool-down. The resistance exercise was the same as the intervention group.
Guo, H (2024)	2 experimental groups and 1 control group were set for this trial. The intervention regimens included tai chi exercise hybrid strength training (TCSG) and strength training (STG). The TCSG and the STG were the experimental groups. All participants in the experimental groups started with a 20-minute warm-up exercise and concluded with a 10-minute cool-down exercise.

	1. Tai chi exercise: The TCSG's tai chi training was structured into 2 cycles. The first cycle (weeks 1-12) focused on learning and consolidating basic tai chi movements, while the second cycle (weeks 13-24) aimed at improving and refining these movements. Each tai chi session lasted 30 minutes and included slow, flowing movements combined with deep breathing exercises to promote physical and mental relaxation.<br><br>2. Strength training: a total of 5 strength training movements were designed, 2 of which were for training the lower body muscles and 3 for training the upper body muscles. The movements to exercise the upper limb muscles were reverse grip curls, seated pull-downs, and bicep curls. The movements to train the muscle strength of the lower limbs were standing leg raises with an elastic band and supine leg lifts with an elastic band. Further, 1 training cycle lasted for 8 weeks, and the whole training had 3 cycles. In the first cycle, participants trained with a light load but many repetitions (from 40% to 60% of 1 repetition maximum [RM] and 12-20 repetitions). A moderate-intensity load with a medium number of repetitions (60%-80% of 1 RM and 5-12 repetitions) was used in the second cycle of training to further enhance the training load. To increase the participants' maximum muscular strength in the third cycle, a greater training load and fewer repetitions (70%-85% of 1 RM and 5-8 repetitions) was used. Participants in the STG performed 4 sets of each movement, while the TCSG completed 2 sets with a 2 ~~to~~ -3 minute rest between each set.<br><br>3. Control Group: the participants in this group were provided with information on sarcopenia treatment and prevention, such as increasing protein intake and engaging in general physical activities.
Sang Jung, W (2019)	Experimental group (EG) participants performed a total of two days of pre-testing and followed by 12 weeks of circuit exercise training sessions, and they then completed two days of post-testing. On the first testing day, all participants had been fasting for more than eight hours before coming to the laboratory. After 30 minutes of rest, body composition and balance were measured. On the second testing day, following a rest day, muscular and pulmonary functioning were measured. EG participants then followed the exercise intervention consisting of a warm-up period and then 25-75-minute sessions (see later for more detail on the variations in time length) three times per week for a total of 12 weeks. The circuit training exercise consisted of 10 movements: walking in place, shoulder press and squat, twist dash, lunge, jumping jacks, kick back, push up, crunch, hip bridge, and bird dog. The program ended with a cool down period of 10 minutes. The main exercise set was performed for 10 minutes followed by five minutes of rest before the next set. In Weeks 1-2, the training session lasted 25 minutes, in Weeks 3-8, it lasted 40 minutes, and in Weeks 9-12, it lasted 55 minutes. The exercise intensity levels ranged from 60% to 80% of the heart rate reserve (HRR).<br><br>Control group (CG) participants maintained their usual physical activity lifestyle for the duration of the study (October 2016–February 2017). In this routine, CG participants were encouraged to check in by telephone twice a week to maintain daily lifestyle (physical activity and dietary intake), and they were taught nutrition education in the lab every four weeks. CG participants underwent the same preintervention and postintervention testing as EG participants.
Ilke Sen, E (2021)	The home-based training program included posture and stretching exercises, strengthening exercises, balance training, and a walking regimen 3 days per week for 3 months. The training program was performed with a light intensity maintained at approximately 10–12 on the Borg Rate of Perceived Exertion scale. Exercises were performed for a gradually increasing duration. At the end of 1 month, the participants performed a 5-min warm-up, 10 min of strengthening exercises, and 10 min of balance exercises, followed by a 5-min cooldown period and 30 min of gait training.<br>The initial warm-up program consists of shoulder, lower back, hip, knee, and ankle stretching exercises and stepping. Exercises were performed in a progressive sequence from the seated position to the standing position. Strength training included shoulder flexion and abduction; elbow flexion; hip flexion, extension, and abduction; knee extension; and ankle dorsiflexion. Resistance was applied using 0.50-kg hand weights for the upper extremities and 0.50- and 1.00-kg ankle cuff weights for the lower extremities. The balance training included standing on the toes, going from a sitting to a standing position, going from a standing to a sitting position, tandem stance, tandem walking, and one-leg stands. As the program progressed, the number of repetitions of each exercise increased, beginning with three repetitions and reaching 10 by Week 8. Ten repetitions were maintained for the rest of the program. Over 2 months, the participants increased the amount of each exercise from one to two sets of 10 repetitions. At the end of the session, they had a cooldown period with muscle relaxation, controlled breathing, and stretching exercises. Participants were advised to walk outside the home and to focus on stability maintenance during slow to moderate walking at least three times a week.

IG: intervention group, CG: control group
